# Tracing the pathways and mechanisms involved in medicinal uses of flaxseed with computational methods and bioinformatics tools

**DOI:** 10.3389/fchem.2023.1276052

**Published:** 2024-01-12

**Authors:** Sravani Joshi, Ruby Srivastava

**Affiliations:** Centre for Cellular and Molecular Biology-CSIR, Hyderabad, India

**Keywords:** flaxseed, hypertension, addictions, Docking, side effects

## Abstract

Pharmacological drugs targeting specific pathways involved in various diseases have seen recent advancement with newer and more efficient emerging drug targets, but these drugs are limited in terms of their side effects and patient adherence. The potential of plant-based diets in the form of functional foods is increasingly being realized as an option to treat and/or prevent several diseases. In this work, we have selected flaxseed (*Linum usitatissimum*), also known as linseed, to study its pharmacological efficacy and proposed mechanisms of action for medicinal purposes. The target genes of linseed with Disease Specificity Index (DSI >0.6) are compared to the associated genes of diabetes mellitus, decrease in appetite, addictive behavior, cardiovascular diseases (CVDs), inflammatory bowel diseases (IBDs), and Polycystic Ovary Syndrome (PCOS), and the selected genes are further evaluated using *in silico* methods. The binding affinity of flaxseed to three common target proteins (CCDC28b, PDCD6IP, and USP34) is assessed by docking and molecular dynamics (MD) simulations. The results show that linseed is safe to use for mutagenic toxicity and other cardiotoxicity measures, but linseed is unsafe for embryotoxicity, hERG toxicity, and cardiac failure. The analysis of the protein–protein interaction (PPI) network, Gene Ontology (GO), and Kyoto Encyclopedia of Genes and Genomes (KEGG) pathways indicates that flaxseed can be used as a medicinal herb for treatment of diabetes mellitus, cardiovascular diseases, IBDs, and PCOS.

## Introduction

Flaxseed or linseed (*Linum usitatissimum* L.) belongs to the Linaceae family, which is used as an edible medicinal herb due to the large amount of high-quality protein and soluble fiber. Flaxseed oil has many health benefits as it has a proper balance of essential fatty acids such as α-linolenic acid (ALA); an omega-3 biological precursor, linoleic acid; an omega-6 fatty acid; and omega-9 fatty acids. Potassium, lecithin, magnesium, 6% mucilage, traces of cyanogenic glycoside linamarin (El-galil and Mohammed, 2021), and vitamins (A, B, D, and E) are present in flaxseed. Flaxseed also includes phytoactive chemicals such as phenolic compounds, terpenoids, pigments, and other naturally occurring antioxidants. Cyclic peptides (cyclopeptides and cyclotides) isolated from plants and animals show various biological functions such as antidiabetic, cardioprotective, and immunosuppressive properties, which can be used to develop many therapeutic agents (Górski et al., 2001; Drygała et al., 2009; Thevenard et al., 2010; Yu et al., 2010; Hu and Xu, 2012; Chang et al., 2013; Fang et al., 2013; Goyal et al., 2014; Boivin et al., 2015). High contents of ALA in flaxseed oil, lignans, dietary fibers, and flaxseed proteins have drawn the attention of the scientific community in exploiting the maximum benefits of flaxseed for medicinal purposes (Marambe and Wanasundara, 2017; Zou et al., 2017; Wu et al., 2019; Yang et al., 2021). Dietary supplementation with flaxseed is beneficial for cardiovascular diseases (CVDs) as use of flaxseed supplement shows antihypertensive action, antiatherogenic effects, lowering of cholesterol, anti-inflammatory action, and inhibition of arrhythmias. Although not well-known, few biological actions of flaxseed are attributed to potential bioactive compounds such as proteins, cyclolinopeptides, and cyanogenic glycosides. The cardioprotective effects of polyunsaturated fatty acids (PUFAs) has been observed in several clinical trials such as DART (Burr et al., 1989), the GISSI-Prevenzione trial (Bang et al., 1971), GISSI-HF (Bang et al., 1976), and JELIS (Gonzalez et al., 2011). Previous studies have shown that the consumption of flaxseed (for 8 to 12 weeks) in the long term reduces blood glucose (Moreira et al., 2022), glycated hemoglobin (Pan et al., 2007; Hasaniani et al., 2019), triglycerides (Torkan et al., 2015), total cholesterol (Toulabi et al., 2022), and blood pressure (Khalesi et al., 2015) in patients with T2DM (Hasaniani et al., 2019) and insulinemia (Rhee and Brunt, 2011). Consumption of flaxseed improves insulin sensitivity in individuals with prediabetes (Javidi et al., 2016) and obesity-related issues (Bongartz et al., 2022). Several studies indicate that the consumption of flaxseed improves glycemic control (Mohammadi-Sartang et al., 2018; Hajiahmadi et al., 2020; Villarreal-Renteria et al., 2022). The acute effects of flaxseed on postprandial hyperglycemia in individuals with T2DM have not been investigated until now (Moreira et al., 2022). Flaxseed is also used as a therapeutic agent in inflammatory bowel disease (IBD), which is a heterogeneous disease in which multiple triggers act simultaneously (Palla et al., 2020). The main targets of IBD are immune dysregulation, polyendocrinopathy, and microbial defects, which is accompanied with symptoms such as abdominal spasms and colic (Xavier and Podolsky, 2007). Even with advance IBD therapeutics, the rate of failure remains high, possibly due to multiple causes of the disease (Duke et al., 2002; Rahimi et al., 2010). The aqueous-methanolic crude extract of Flaxseed (Fs.Cr) has improved the severity in the mouse model of colitis by reducing mediators of inflammation (myeloperoxidase and cytokines) and inducers of oxidative stress (Palla et al., 2016). Flaxseed has been reported to have antihyperglycemic properties without any reported severe side effects (Mani et al., 2011; Parikh et al., 2019). The functional compounds of flaxseed provide several health benefits related to improvement in disease in individuals with metabolic syndrome (Hutchins et al., 2013; Shayan et al., 2020; Yari et al., 2020). Consumption of 30 g of flaxseed added to a bread recipe or in a 50-g glucose challenge reduces the blood glucose area under the curve (AUC) for more than 2 h in healthy young adults (Dahl et al., 2005; Vuksan et al., 2017). Lignan in flaxseed reduces androgen levels in men with prostate cancer (Demark-Wahnefried et al., 2008). Polycystic Ovary Syndrome (PCOS) is a chronic endocrinopathy which affects few women of reproductive age (Nowak et al., 2007, Yumiceba et al., 2020). Persistent anovulation and hyperandrogenism are the characteristics of PCOS, which results in high blood sugar and type 2 diabetes (T2D) at an early stage, dyslipidemia, cardiovascular disease, and infertility (Franks and Hardy, 2020). The most important PCOS candidate genes are those that encode for molecules involved in androgen synthesis, transportation, and control the secretion and activity of insulin receptors, signaling cascade proteins, and growth factors (Wang et al., 2020).

The main genomic investigations are focused on the genes involved in metabolism and biosynthetic pathways, reproductive function, signaling pathways, transportation and development, cellular senescence, and other biological processes (Deswal et al., 2019). Drugs such as metformin, clomiphene citrate, and glucocorticoids and aromatase inhibitors like anastrozole are used for the treatment of PCOS (Badawy and Elnashar, 2011), which cause numerous side effects such as nausea, discomfort in the abdomen, and vaginal bleeding (Ndefo et al., 2013). So herbal drugs as phytoestrogen (isoflavonoids, flavonoids, stilbenes, and lignans), which are abundant in soy isoflavones and flaxseed lignans (Mehraban et al., 2020), are recommended as an alternative.

## Materials and methods

Conventional methods to discover and characterize potential bioactive peptides from food proteins are time-consuming and costly, so *in silico* approaches are used to predict the potential bioactive peptides from various food proteins (Udenigwe et al, 2012; Poustforoosh et al., 2023, Ji et al., 2020). Computational resources are cost-effective and provide a significant amount of information in a limited time span for herbal complexes. Computational tools can predict the physicochemical and biological properties of herbal medicine in less time and accurately predict the possible target–drug interactions. As flaxseed has multifactorial activities and no proper detailed information related to the pharmacodynamics, mechanism of action, absorption, and toxicity on flaxseed is reported, we carried out a detailed computational work to study the multifarious activities of flaxseed. At first, the physicochemical and absorption, distribution, metabolism, excretion, and toxicity (ADMET) properties of the main constituent (α-linolenic acid) of flaxseed are studied. Then, the potent targets for linseed are identified. After determining the disease-associated genes, the protein–protein interaction (PPI) network, Gene ontology (GO), and Kyoto Encyclopedia of Genes and Genomes (KEGG) Pathway were analyzed. Docking studies are carried out for the three target–flaxseed complexes, and then the molecular dynamics (MD) simulations for the three target–flaxseed complexes are carried out with a Galaxy platform using open-source GROMACS tools (Galaxy, 2023). The results were analyzed by Root Mean Square Deviation, Root Mean Square Fluctuation, and principal component analysis (PCA) plots.

### Physicochemical and ADMET properties of flaxseed (α-linolenic acid)

Flaxseed consists of chemical compounds with specific biological activity and functional properties: PUFAs, omega-3 family, soluble dietary fibers, lignans, proteins, and carbohydrates. The major composition of flaxseed includes fatty acids (α-linolenic acid, linoleic acid, oleic acid, stearic acid, and palmitic acid) (Bernacchia et al., 2014). ALA comprises approximately 55% of the total fatty acid content of flaxseed fatty acids (McCullough et al., 2011). Experimental trials showed that ALA-rich diets reduced the occurrence of both fatal and non-fatal myocardial infarction (de Lorgeril et al., 1994; Albert et al., 2005), cardiac arrhythmias (Albert et al., 2005), and atherosclerotic lesions (Albert et al., 2005; Tziomalos et al., 2008). The SMILES notation for the main constituent of linseed: ligand (DB00132, α-linolenic acid) is obtained from the DrugBank (https://go.drugbank.com/drugs/DB00132) for predicting the physicochemical properties and toxicity (mutagenic effect, irritant effect, tumorigenicity, and effect on the reproductive system) effects. Various conformational structures of α-linolenic acid are generated from the PDB file (DB00132), and then all the structures are optimized by the wB97XD (Chai and Head-Gordon, 2008)/6–31G (d,p) method with G09 software suites (Frisch et al., 2009). The integral equation formalism-polarized continuum model (IEF-PCM) is utilized with water as a solvent (Marenich et al., 2009). Finally, the lower minima structure with no negative frequency is considered for further studies. The SMILES notation of α-linolenic acid is used as the input to study the physicochemical properties and toxicity measures by Molinspiration software (www.molinspiration.com) (Molinspiration Chemoinformatics software, 2023) and Osiris software program (Osiris property explorer (www.organicchemistry.org/prog/peo/)), (Osiris property explorer, 2023) respectively. Molinspiration software is used to predict the physicochemical properties including logP, molecular polar surface area (PSA), and the descriptors from Lipinski “Rule of 5.” The Osiris software program is used to predict various drug-relevant properties. The mutagenic toxicity-related results are color-coded. Properties with high risks of undesired effects like mutagenicity or poor intestinal absorption are shown in red. The green color indicates drug-conformation behavior, whereas yellow color indicates mild risk.

The cardioToxCSM (Iftkhar et al., 2022) webserver was used to study six types of cardiotoxicity outcomes in flaxseed: arrhythmia, cardiac failure, heart block, hERG toxicity, hypertension, and myocardial infarction. The cardioToxCSM is a machine learning (ML)-based webserver which is developed by using the concept of graph-based signatures, molecular descriptors, toxicophore matchings, and molecular fingerprints. The datasets are internally and externally validated via different cross-validation schemes and low-redundancy blind sets, respectively. The embryoTox (Aljarf et al., 2023) webserver is used to predict and classify molecules which are likely to be safe to use during pregnancy in women. The webserver is trained and validated using *in vitro* bioactivity data of over 700 small molecules. embryoTox utilizes a graph-based signature representation of the chemical structure with teratogenicity effects.

### Identification of flaxseed potential targets

The ChEMBL database (https://www.ebi.ac.uk/chembl) is utilized to identify the potential targets of linseed. “Flaxseed” or linseed or “*Linum usitatissimum*” or SMILES notation of α-linolenic acid are used as the keywords. A total of 150 potential targets for *Homo sapiens* were obtained from the target summary in the compound’s report card with similar structures and extensive search.

### Determination of disease-associated genes

The associated genes for various diseases are determined using the DisGeNET database (https://www.disgenet.org). As flaxseed is used as therapeutic agents for cardiovascular diseases (Rodriguez-Leyva et al., 2010), DM (Javidi et al., 2016), addictive behavior (Prior et al., 2015), IBD (Palla et al., 2020), and PCOS (Nowak et al., 2007), the associated genes with *Disease Specificity Index* (DSI) greater than 0.6 for *Homo sapiens* are compared. Ten potential target genes were identified, out of which three potential target proteins which are common to cardiovascular diseases, DM, IBD, and PCOS, were selected for further studies. Therefore, no common target genes are identified for addictive behavior.

### Protein–protein interaction network, enrichment of Gene Ontology, and Kyoto Encyclopedia of Genes and Genomes pathway

The STRING database v11.5 (https://stringdb.org/) (String database V11.5, 2023) was used to find the PPI network of the potential targets. The suggested pathways are based on the known interactions (previously reported studies), predicted interactions (e.g., gene co-occurrence), or various other reports (e.g., text mining) to investigate the molecular functions and biological processes. The GO and KEGG (https://www.genome.jp/kegg/) analyses were carried out to find the pathways modulated by flaxseed targets. GO and KEGG enrichment analyses are commonly used to analyze genes (Chen et al., 2017). GO, a database from the Gene Ontology Consortium, with a set of dynamic administered vocabularies was used to understand the roles of genes and proteins in cells, thereby extensively characterizing genes and gene products by biological process (BP), cellular component (CC), and molecular function (Ashburner et al., 2000; The Gene Ontology Consortium, 2023) in living organisms. KEGG, a manually curated database, is used to integrate various biological objects that are divided into systems, genomes, and other health-related resources (Kanehisa et al., 2023). The core pathways revealed by KEGG enrichment analysis show the core pathways, and the connections between these basic genes can further help understand the key roles of these genes.

### Docking and MD simulations

Once the key roles of target genes are identified, these common target genes are selected for docking and MD simulations with flaxseed (α-linolenic acid). Molecular docking in drug discovery plays a vital role as it includes structure–activity relationship (SAR), lead optimization, finds potential leads through virtual screening, and provides binding measures to facilitate predictions for inhibitors (Schleinkofer et al., 2006). The 3D structures of the selected three target proteins are obtained from the EBI AlphaFold2 database (Jumper et al., 2021) (EMBL-EBI alphafold, 2023). These three target proteins are CCDC28b (Q9BUN5), PDCD61P (UniProt ID: Q8WUM4), and USP34 (UniProt ID: Q9P2H5) with source species as *Homo sapiens*. The lower energy minima structure with no negative frequency of flaxseed (α-linolenic acid) is selected for the target–ligand interaction. The binding energies of target–flaxseed complexes are calculated with AutoDock tools (Morris et al., 2009). The output structures of these docked structures are used as input for energy minimization and MD simulations with the Galaxy webserver platform (Galaxy webserver; usegalaxy.org). The Galaxy platform is used for high-throughput molecular dynamics simulation to study protein–ligand interactions using the open-source GROMACS tools. For protein topology, the TIP3P model with an AMBER99SB force field is used. Ligand topology is generated by the GAFF force field with default BCC charge method (0 charge, multiplicity 1). The simulation box is created with box dimensions of 1.0 nm and triclinic shape for energy minimization. The system is charged (depending on the pH) with water as a solvent, and it also adds sodium or chloride ions (replacing existing water molecules) as per requirement for neutralization. The EM tolerance = 1,000, and 50,000 steps are considered for energy minimization. Energy minimization (EM) is used to relax the structure, and any steric clashes or unusual geometry are removed. The equilibration of the solvent around the solute (i.e., the protein) is performed in two steps; the equilibration under an NVT (or isothermal–isochoric) ensemble, followed by an NPT (or isothermal–isobaric) ensemble. For NVT and NPT calculations, the following parameters are used*: bond constraints (constraints)—all bonds*, *temperature/K—300*, *step length in ps—0.001*, and *number of steps that elapse between saving data points (velocities*, *forces*, and *energies)—1,000* and *number of simulation steps—50000. For production simulation, the parameter settings are as follows: ensemble—NPT, temperature /K—300, step length in ps—0.001, number of steps that elapse between saving data points (velocities*, *forces*, and *energies)—1,000*, *and number of simulation steps—100000.* In this manner, the simulation will run for 1 million steps, with the total length of 1 ns. This post-study is repeated 20 times for 20-ns MD simulations, and then the final plots for Root Mean Square Deviation, Root Mean Square Fluctuation, and PCA are reported independently for target–flaxseed interactions. The Root Mean Square Deviation is used to calculate the difference between a protein’s backbone Cα atoms (at final position) compared to its original conformational structure. The deviation during protein simulation is used to determine the stability of its conformational structure (Jiang et al., 2019). Root Mean Square Deviation is used to examine the free-energy landscape (FEL) and display the compactness and conformational stability of proteins during the dynamic period. Root Mean Square Fluctuation is a crucial measure that is used to assess how much an atom group deviates from its distinct place in a constituted system during an MD simulation (Jiang et al., 2019). The obtained results from docking and MD simulations are visualized with PyMOL software (Schrödinger DeLano, 2020).

## Results

The chemical structures, SMILES notation, physicochemical parameters, and various toxicity measurements of flaxseed (α-linolenic acid) are given in Table 1. The oral active nature of a drug complex is predicted by Lipinski’s ‘‘rule of 5’’ (Lipinski et al., 1997) which follows i) the molecular weight (MW) < 500, ii) the calculated octanol/water partition coefficient (log P) < 5, iii) fewer than five hydrogen bond donors (HBDs) (OH and NH groups), and iv) less than 10 hydrogen bond acceptors (HBAs) (notably, N and O). During drug discovery, lipophilicity and molecular weight are often increased in order to improve the affinity and selectivity of the drug candidate. Hence, sometimes it is difficult to maintain drug-likeness (i.e., RO5 compliance) during hit and lead optimization. In flaxseed, it is seen that one parameter violates the Lipinski’s ‘‘rule (log P) > 5.” If the calculated logP (logarithm of partition coefficient) values for the LOBs are between 2 and 5 (from ALOGpS, http://www.vcclab.org), it is suitable for both oral and topical administrations. The topological polar surface area (TPSA) is interconnected to the hydrogen-bonding potential of the complexes (Deconinck et al., 2007). The complexes with TPSA values >140 Å^2^ or more showed poor intestinal absorption (Daina et al., 2017), and TPSA <90 Å^2^ is required to penetrate the blood−brain barrier, and thus, act on receptors in the CNS. The TPSA of flaxseed is 37.30 Å^2^, which is suitable to penetrate the BBB. The bioactivity score of more than 0.00 for flaxseed indicates considerable biological activities. Since the inappropriate ADMET properties of drugs prohibit their usage at the clinical level, the toxicity risks (mutagenicity, tumorigenicity, irritation, and reproductive effect) are also predicted for flaxseed (α-linolenic acid). The results show that flaxseed is safe to use for mutagenic toxicity (green tick), which is also validated by drug-score prediction. As toxicity is the main issue related to the drugs, computational approaches with quantitative structure–activity relationship (QSAR) models and ML methods are used to identify six types of toxicity as cardiac toxicity outcomes: arrhythmia, cardiac failure, heart block, hERG toxicity, hypertension, and myocardial infarction efficiently and accurately. The cardiotoxicity results show that it is harmful to use flaxseed in hERG toxicity and cardiac failure, while it is safe to use in arrhythmia, heart block, hypertension, and myocardial infarction. The embryoTox results indicate that it is unsafe to use flaxseed during pregnancy in women (Table 1).

**TABLE 1:** Chemical structure of α-linolenic acid, SMILES notation, physicochemical parameters, and toxicity measures for flaxseed. Abbreviations: mutagenic effect (MUT), irritant effect (IRRI), tumorigenicity (TUM), and effects on the reproductive system (REP). Green tick indicates that the complex is safe to use. SMILES notation for α-linolenic acid is taken from DB00132, and the chemical structure is optimized by the wB97XD/6–31G (d,p) method with G09 software suites. Physicochemical parameters are taken from Molinspiration software, and the Osiris software is used to estimate MUT, IRRI, TUM, and REP. The cardiotoxicity and embryotoxicity is predicted by CardioToxCSM and EmbryoTox webserver respectively.

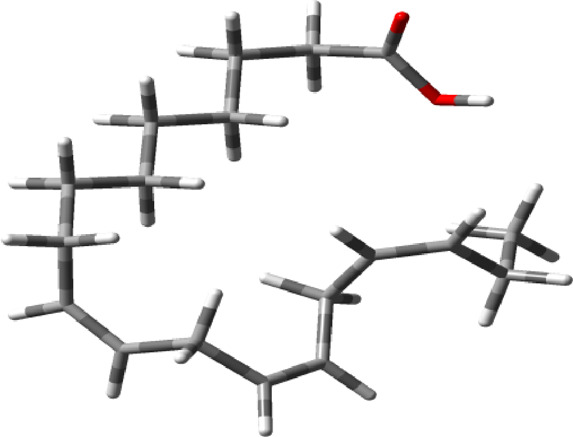 Chemical structure of α-linolenic acid		CC/C=C\C/C=C\C/C=C\CCCCCCCC(=O)O (SMILE notation)								
Physicochemical parameters		miLogP		5.84		Volume		306.47		
		TPSA		37.30 Å^2^		Solubility		−4.09		
		MW		278.44 (g/mol)		Drug likeness		−20.74		
		nON		2		Drug Score		−0.25		
		nOHNH		1		nAtoms		20		
		Nrotb		13						
Toxicity measures										
Cardiotoxicity	Arrhythmia		Cardiac failure		Heart block		hERG toxicity		Hypertension	Myocardial infarction
Measures	Safe		Toxic		Safe		Toxic		Safe	Safe
Mutagenic toxicity	MUT		TUM		IRR		REP		—	—
Measures	**✓**		**✓**		**✓**		**✓**		—	—
Embryotoxicity	Safety profile		Confidence level		—					
Measures	Unsafe		High							

The genes associated with the cardiovascular diseases (C0007222)-517 genes, diabetes mellitus (C0011849)-516 genes, inflammatory bowel disease (C0021390)-500 targets, addiction behavior (C0085281)-332 genes, and PCOS-297 are obtained from the DisGeNET database, and then these genes are compared to the flaxseed genes with the help of the DisGeNET database, with DSI >0.6 (*Homo sapiens*). Ten potential target genes were identified, out of which three common potential target genes (CCDC28b, PDCD6IP, and USP34) were selected, which are most effective for cardiovascular diseases, diabetes mellitus, IBD, and PCOS (Table 2).

**TABLE 2:** Target proteins of flaxseed associated with cardiovascular diseases (C0007222), diabetes mellitus (C0011849), inflammatory bowel disease (C0021390), and polycystic ovary syndrome for *Homo sapiens* (organism). The selected target genes with disease specificity index > 0.6 (*Homo sapiens*) are obtained from the DisGeNET database.

Entry	Entry name	Gene name
Q96G61	NUD11	*NUDT11*, *APS1*, and *DIPP3B*
P54760	EPHB4	*EPHB4*, *HTK*, *MYK1*, and *TYRO11*
P00519	ABL1	*ABL1*, *ABL*, and *JTK7*
O60885	BRD4	*BRD4* and *HUNK1*
Q13772	NCOA4	*NCOA4*, *ARA70*, *ELE1*, and *RFG*
P03372	ESR1	*ESR1*, *ESR*, and *NR3A1*
Q9NWZ3	IRAK4	*IRAK4*
Q16204	CCDC6	*CCDC6*, *D10S170*, and *TST1*
A6NNY8	UBP27	*USP27X*, *USP22L*, and *USP27*
Q04609	FOLH1	*FOLH1*, *FOLH*, *NAALAD1*, *PSM PSMA*, and *GIG27*
O75340	PDCD6	*PDCD6* and *ALG2*
O15294	OGT1	*OGT*
Q9Y3Q0	NALD2	*NAALAD2*

**FIGURE 1:**
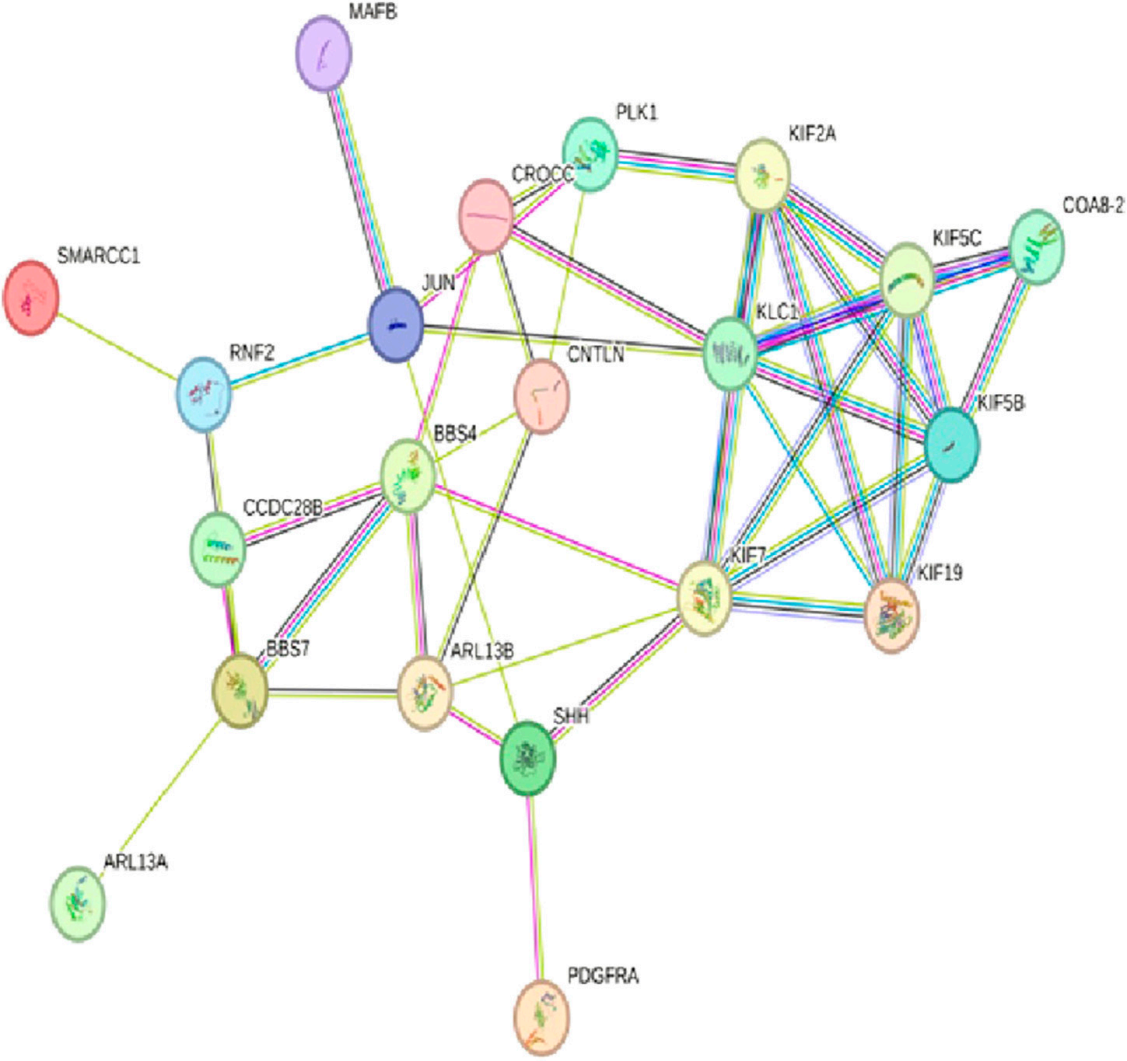
The PPI network of CCDC28b pathways engaged in PMID:29445114: kinesin 1 regulates cilia length through an interaction with the Bardet–Biedl syndrome using the STRING database v11.5 (https://stringdb.org/).

**FIGURE 2:**
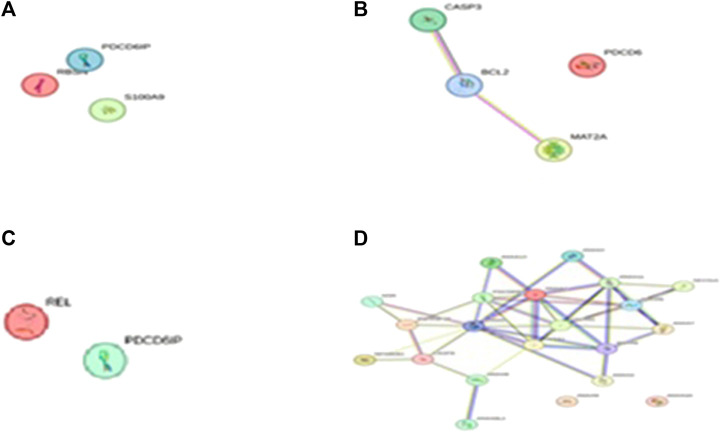
The PPI network of PDCD6IP pathways engaged in **(A)** **(D)** PMID:25644331 Programmed cell death 6 interacting protein (PDCD6IP) and Rabenosyn-5 (ZFYVE20) are potential urinary biomarkers for upper gastrointestinal cancer. String database V11.5 (https://stringdb.org/) is used to predict the PPI network pathways, **(B)** PMID:23777424 A functional insertion–deletion polymorphism in the promoter of PDCD6IP is associated with the susceptibility of hepatocellular carcinoma in a Chinese population, and **(C)** PMID:22369209: PDCD6 is an independent predictor of progression free survival in epithelial ovarian cancer.

**FIGURE 3:**
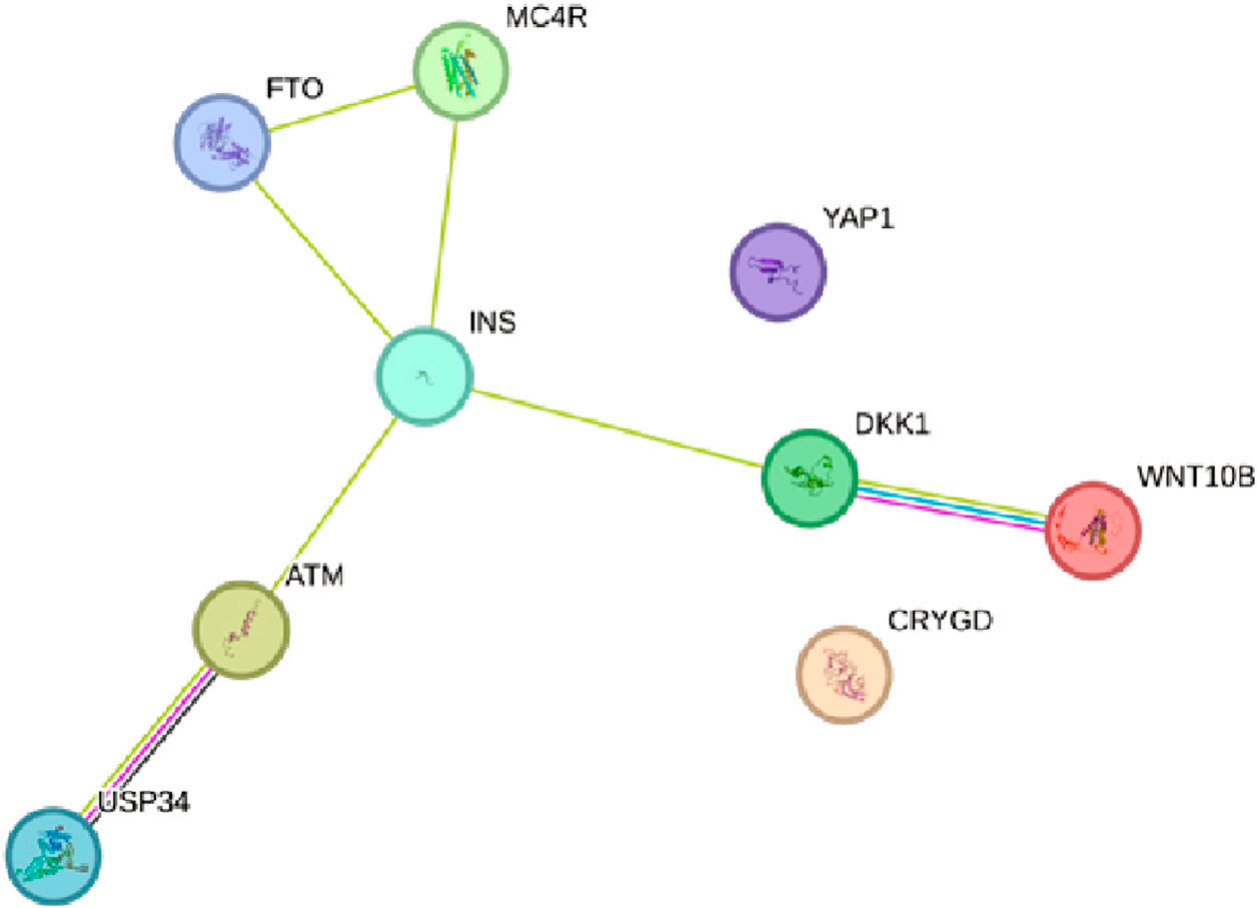
PPI network of the USP34 pathways engaged in polycystic ovary syndrome using the STRING database v11.5 (https://stringdb.org/).

After determining the potential targets of flaxseed, they are assessed for its association in various diseases. One of the targets CCDC28b with a PPI network ID PMID:29445114: kinesin 1 regulates cilia length through an interaction with the Bardet–Biedl syndrome, associated to the Bardet–Biedl syndrome disease which is a new player in hypertension and other cardiovascular risk factors such as obesity and renal abnormalities (Zhao and Rahmouni, 2022). Complications of obesity can include type 2 diabetes, high blood pressure (Caligiuri et al., 2016), (hypertension), and abnormally high cholesterol levels (hypercholesterolemia) (Jiamset and Hanprasertpong, 2016). The PPI network analysis for CCDC28b is given in Figure 1. Other targets, PCDC6 and PCDC6IP, are associated with PMID:35396512:MAT2A, which facilitates PDCD6 methylation and promotes cell growth under glucose deprivation in cervical cancer. PMID:25644331; programmed cell death 6-interacting protein (PDCD6IP) and Rabenosyn-5 (ZFYVE20) are potential urinary biomarkers for upper gastrointestinal cancer (Sarosiek et al., 2016), PMID:23777424; a functional insertion–deletion polymorphism in the promoter of PDCD6IP is associated with the susceptibility of hepatocellular carcinoma in a Chinese population and PMID:22369209; PDCD6 is an independent predictor of progression-free survival in epithelial ovarian cancer (Figure 2). Previous studies during the last decade indicate significant epidemiological evidence to firmly connect certain cancers, especially breast, colorectal, endometrial, hepatic, pancreatic, and kidney, with type 2 DM (Zhan et al., 2010; Larsson and Wolk, 2011; Szablewski, 2014), though the mechanisms of cancer development related with DM remain unclear. Recent studies showed that there is an association between potential urinary biomarkers for upper gastrointestinal cancer and CVD, but the pathophysiological mechanisms underlying these associations are unclear (Jovani et al., 2022). There is also a link stating that diabetes may increase the risk of gastric cancer through shared risk factors including obesity, insulin resistance, hyperinsulinemia, and smoking. Hyperglycemia, even before the clinical diagnosis of diabetes, may predict gastric cancer in some epidemiological *in vitro* and *in vivo* studies (Tseng and Tseng, 2014). IBD, GI cancers, and celiac disease have shown variations in the urinary metabolomics which are associated with possible GI dysbiosis, but there is no study which has systematically assessed the GI microbiota profile simultaneously. The third target gene, USP34 PMID:25975428, has an association with polycystic ovary syndrome. (Zhao et al., 2015) PCOS is a kind of reproductive and metabolic disorder that is characterized by hyperandrogenism and insulin resistance and has affected mostly reproductive-aged women in Caucasia and China (Azziz et al., 2004; Goodarzi and Azziz, 2006; Li et al., 2013) (Figure 3). As PCOS is a heterogeneous endocrine disorder which is characterized by hyperandrogenism, ovulatory dysfunction, and polycystic ovaries, recent epidemiological findings showed that women with PCOS have high chances to develop certain cancer types due to their shared metabolic and endocrine abnormalities.

The GO analysis for CCDC28b show heart looping, heart morphogenesis, and heart development related to cardiovascular diseases (Table 3). Another PPI network GO analysis for PDCD6 and PDCDIP showed the vascular endothelial growth factor receptor-2 signaling pathway, negative regulation of the biological process, and negative regulation of phospholipase A2 activity which is related to cancer and other related diseases (Table 4). The GO terms for USP34 indicate a response to oxygen-containing compound and regulation of fat cell differentiation, which are closely related to diabetes mellitus and cardiovascular diseases (Table 5). The KEGG pathway analysis predicts hsa:79140 KEGG genes for CCDC28b, hsa:10016 and hsa:10015 for PDCD6, and hsa:9736 KEGG genes for USP34.

**TABLE 3:** The Gene Ontology (GO) terms of biological processes with a minimum false discovery rate for the CCDC28b PPI network using the STRING database v11.5 (https://stringdb.org/).

#Term id	Term description
GO:0006996	Organelle organization
GO:0007017	Microtubule-based process
GO:0120036	Plasma membrane-bound cell projection organization
GO:0016043	Cellular component organization
GO:0044782	Cilium organization
GO:0032984	Protein-containing complex disassembly
GO:1905515	Non-motile cilium assembly
GO:0001947	Heart looping
GO:0048562	Embryonic organ morphogenesis
GO:0097499	Protein localization to the non-motile cilium
GO:0022411	Cellular component disassembly
GO:0048598	Embryonic morphogenesis
GO:0007018	Microtubule-based movement
GO:0007098	Centrosome cycle
GO:0051656	Establishment of organelle localization
GO:0009653	Anatomical structure morphogenesis
GO:0009887	Animal organ morphogenesis
GO:0003007	Heart morphogenesis
GO:0007389	Pattern specification process
GO:0033365	Protein localization to the organelle
GO:0045444	Fat cell differentiation
GO:0008104	Protein localization
GO:0070925	Organelle assembly
GO:0032434	Regulation of proteasomal ubiquitin-dependent protein catabolic process
GO:0000226	Microtubule cytoskeleton organization
GO:0007507	Heart development
GO:0060271	Cilium assembly
GO:0048546	Digestive tract morphogenesis
GO:0007010	Cytoskeleton organization
GO:0007423	Sensory organ development
GO:0035617	Stress granule disassembly
GO:0097500	Receptor localization to the non-motile cilium
GO:0098971	Anterograde dendritic transport of a neurotransmitter receptor complex
GO:0051650	Establishment of vesicle localization
GO:0007399	Nervous system development
GO:0001654	Eye development
GO:0099641	Anterograde axonal protein transport
GO:0035239	Tube morphogenesis
GO:0010457	Centriole–centriole cohesion
GO:0007224	Smoothened signaling pathway
GO:0060021	Roof of mouth development
GO:0009798	Axis specification
GO:0060322	Head development
GO:0048557	Embryonic digestive tract morphogenesis
GO:1903008	Organelle disassembly
GO:0007019	Microtubule depolymerization
GO:0048468	Cell development

**TABLE 4:** The Gene Ontology (GO) terms of biological processes with a minimum false discovery rate for the PDCD6IP PPI network using the STRING database v11.5 (https://stringdb.org/).

#Term id	Term description
GO:0016050	Vesicle organization
GO:0043086	Negative regulation of catalytic activity
GO:1900138	Negative regulation of phospholipase A2 activity
GO:0016192	Vesicle-mediated transport
GO:0050790	Regulation of catalytic activity
GO:0007032	Endosome organization
GO:0010595	Positive regulation of endothelial cell migration
GO:0006810	Transport
GO:0010035	Response to the inorganic substance
GO:0010256	Endomembrane system organization
GO:0036324	Vascular endothelial growth factor receptor-2 signaling pathway
GO:0050819	Negative regulation of coagulation
GO:0051179	Localization
GO:0048519	Negative regulation of the biological process
GO:1903551	Regulation of the extracellular exosome assembly
GO:0051239	Regulation of the multicellular organismal process
GO:1900004	Negative regulation of serine-type endopeptidase activity
GO:0031340	Positive regulation of vesicle fusion
GO:0052548	Regulation of endopeptidase activity
GO:0006900	Vesicle budding from a membrane
GO:0017157	Regulation of exocytosis
GO:0009611	Response to wounding
GO:0045921	Positive regulation of exocytosis
GO:0061024	Membrane organization

**TABLE 5:** The Gene Ontology (GO) terms of biological processes with a minimum false discovery rate for the USP34 PPI network using the STRING database v11.5 (https://stringdb.org/).

#Term id	Term description
GO:0032526	Response to retinoic acid
GO:0030177	Positive regulation of the Wnt signaling pathway
GO:0045598	Regulation of fat cell differentiation
GO:1901700	Response to the oxygen-containing compound
GO:0007267	Cell–cell signaling
GO:0060828	Regulation of the canonical Wnt signaling pathway
GO:0071300	Cellular response to retinoic acid
GO:0016055	Wnt signaling pathway
GO:2000252	Negative regulation of feeding behavior
GO:0060070	Canonical Wnt signaling pathway
GO:0090263	Positive regulation of the canonical Wnt signaling pathway
GO:0009967	Positive regulation of signal transduction
GO:0009888	Tissue development
GO:0051716	Cellular response to stimulus
GO:1901701	Cellular response to the oxygen-containing compound
GO:0060429	Epithelium development
GO:0090335	Regulation of brown fat cell differentiation

**FIGURE 4:**
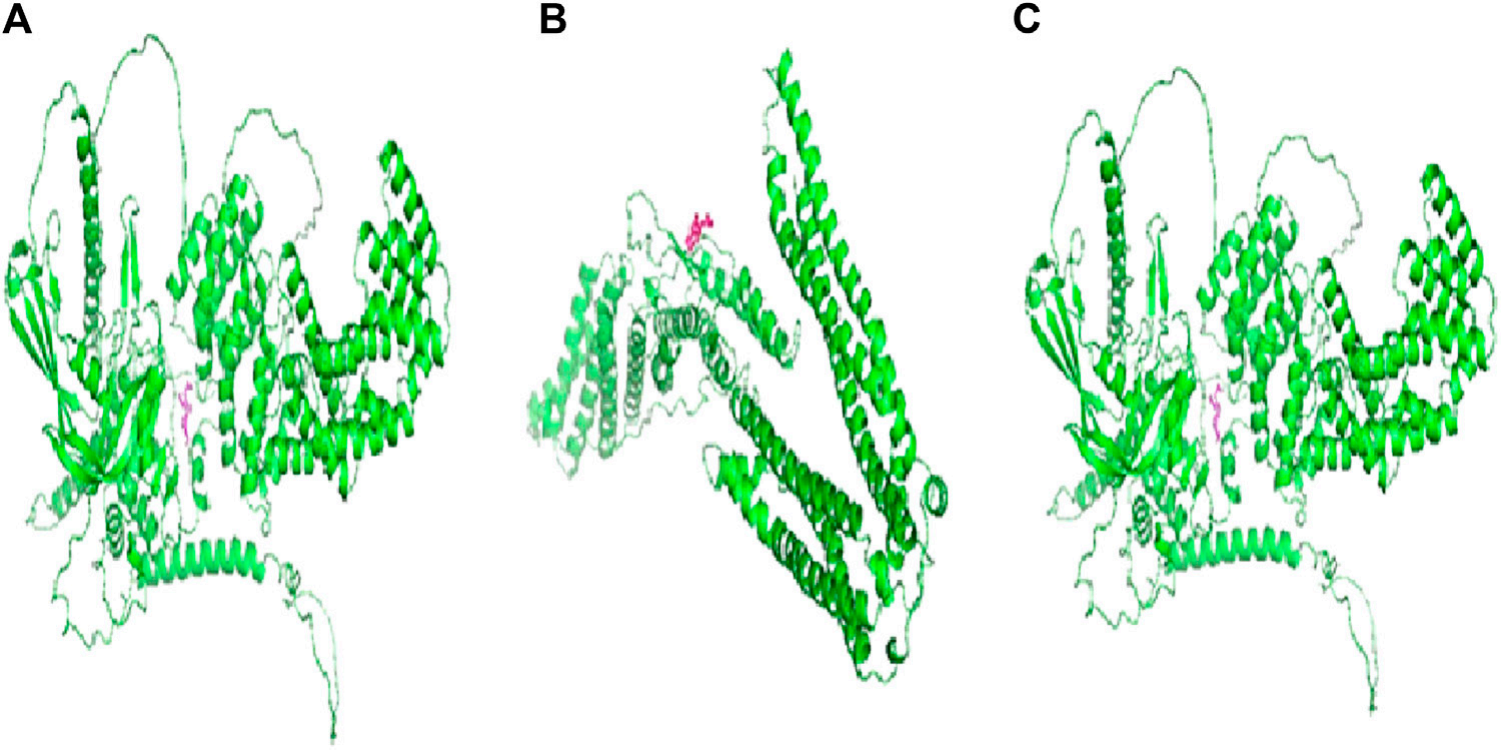
Docking structures and binding energies of **(A)** CCDC28b–flaxseed (BE –2.30 kcal/mol), **(B)** PDCD6IP–flaxseed (BE –2.05 kcal/mol), and **(C)** USP34–flaxseed (BE –4.52 kcal/mol) complexes. AutoDock software tools are used for calculating docking and binding energies for the studied target–flaxseed complexes. The flaxseed (α-linolenic acid) is highlighted with a magenta color.

**FIGURE 5:**
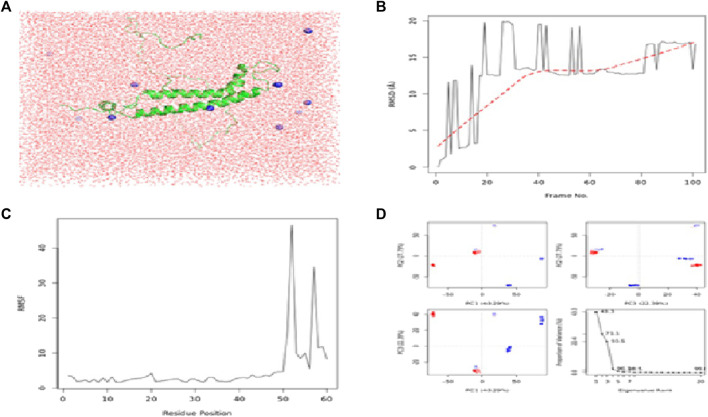
Results of MD simulations for CCDC28b–flaxseed interactions. **(A)** MD simulation structure; **(B)** Root Mean Square Deviation plot; **(C)** Root Mean Square Fluctuation plot; and **(D)** PCA plot. MD simulations for the CCDC28b–flaxseed complex are carried out by the Galaxy platform using open-source GROMACS tools.

**FIGURE 6:**
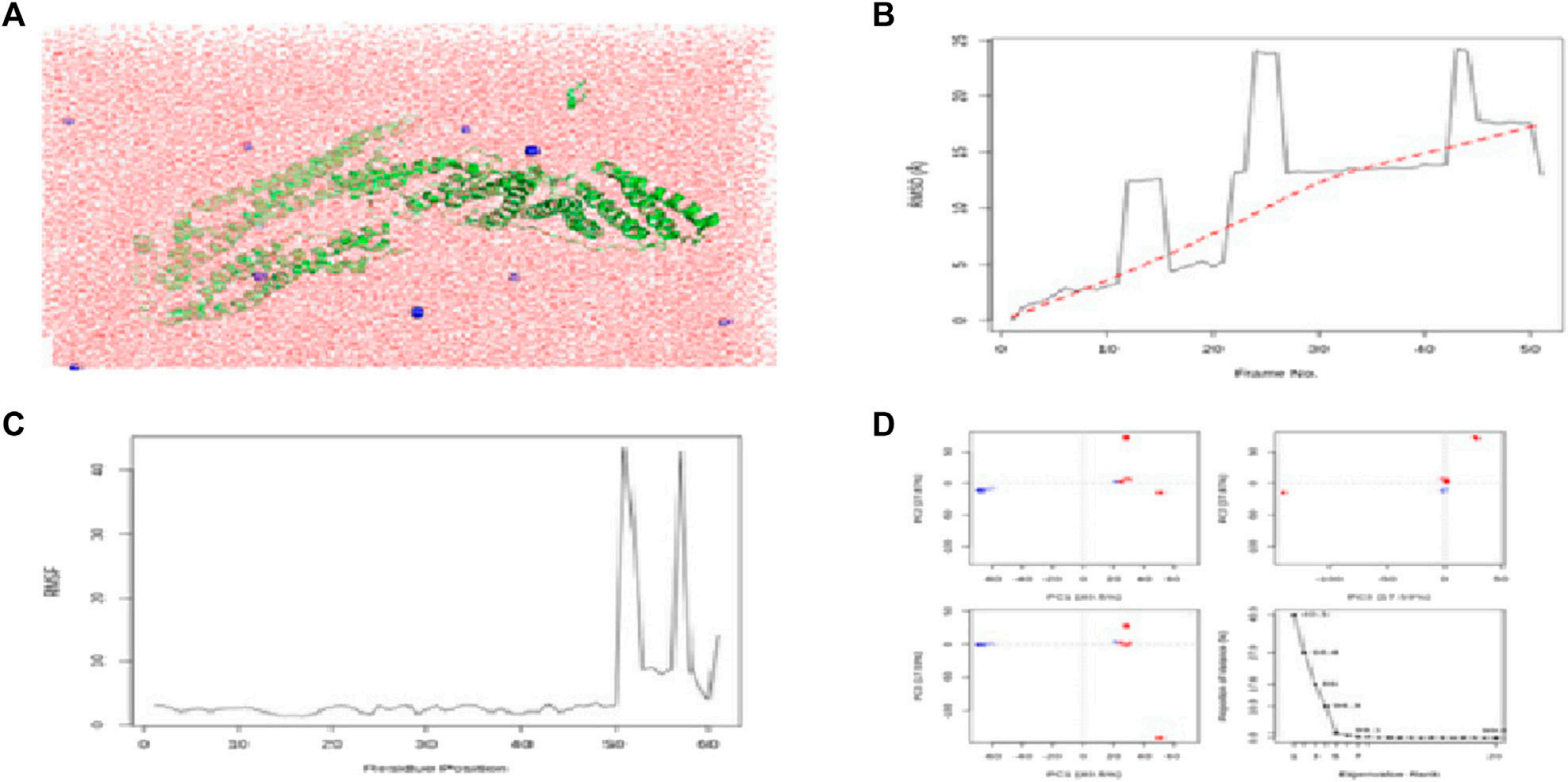
Results of MD simulations for PDCD6IP–flaxseed interactions. **(A)** MD simulation structure; **(B)** Root Mean Square Deviation plot; **(C)** Root Mean Square Fluctuation plot; and **(D)** PCA plot. MD simulations for the PDCD6IP–flaxseed complex are carried out by the Galaxy platform using open-source GROMACS tools.

**FIGURE 7:**
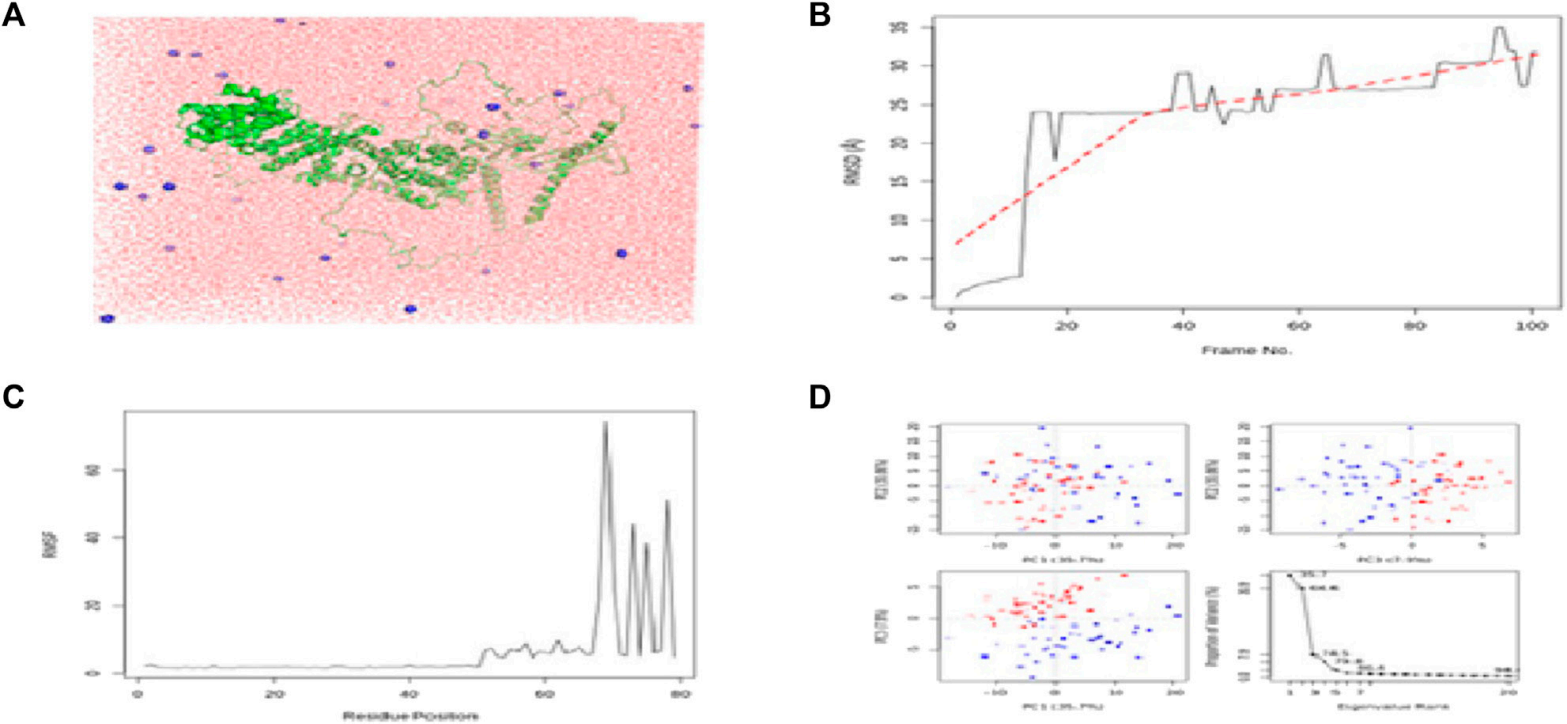
Results of MD simulations for USP34–flaxseed interactions. **(A)** MD simulation structure; **(B)** Root Mean Square Deviation plot; **(C)** Root Mean Square Fluctuation plot; and **(D)** PCA plot. MD simulations for the USP34-flaxseed complex are carried out by the Galaxy platform using open-source GROMACS tools.

The calculated binding energies of these three protein–ligand complexes CCDC28b–flaxseed, PDCD6IP–flaxseed, and USP34–flaxseed, are −2.30 kcal/mol, −2.05 kcal/mol, and −4.52 kcal/mol, respectively. The binding energy is lower for the USP34–linseed complex (Figure 4). These output structures are used as input for MD simulation studies for CCDC28b–flaxseed, PDCD6IP–flaxseed, and USP34–flaxseed complexes. The parameters used for MD simulations in the Galaxy webserver are set with the step length of 0.001 ps and 100,000 steps with 300 K temperature. The Root Mean Square Deviation plot, Root Mean Square Fluctuation, and PCA cluster plot for CCDC28b–linseed, PDCD6IP–linseed, and USP34–linseed complexes are shown in Figure 5, Figure 6, and Figure 7, respectively. Root Mean Square Deviation and Root Mean Square Fluctuation are calculated to check the stability and conformation of the ligand during the simulations. Root Mean Square Deviation is a quantitative measurement used to check the stability of protein–ligand complexes (Kulkarni et al., 2022). Root Mean Square Deviation indicates any changes in the atomic position from the initial structure. The initial increase in the Root Mean Square Deviation for all the three protein–ligand complexes shows system adaptability. However, the flat Root Mean Square Deviation after that shows that the protein conformation has not changed much, and finally, the small increase in Root Mean Square Deviation shows that there is not much deviation of the protein from its original conformation, which is seen in CCDC28b–linseed and USP34–linseed complexes. Root Mean Square Fluctuation measures the average deviation of a protein residue over time from a reference position which is basically the time-averaged position of the protein. Root Mean Square Fluctuation is used to analyze how (much or least) the portions of protein structures are fluctuating. The higher Root Mean Square Fluctuation values at the other end of the graph are most likely the loop regions with more conformational flexibility, where no well-defined structure was observed. All the three (target–linseed) complexes showed the higher Root Mean Square Fluctuation values with time. This will allow a connection with experimental spectroscopic techniques to detect the secondary structure of a protein. The ligand-binding-induced correlated motions are assessed by PCA (Kulkarni et al., 2022; Kumar et al., 2023). The first few eigenvectors control the overall important motion of the protein (Parate et al., 2021). It is anticipated that the resulting plot will indicate higher eigenvalues for the first few vectors, and the most significant motions are contained in the first, second, and third eigenvectors. The PCA results obtained from the Galaxy server indicate the first three eigenvectors which is 43.29%, 27.79%, and 22.39% for the CCDC28b–linseed complex; 40.5%, 27.87%, and 17.59% for PDCD6IP-linseed; and 35.7%, 30.86%, and 7.9% for USP34–linseed complexes, indicating the better integrity of the three target–ligand complexes.

The computational investigations of the potential targets using bioinformatics databases predict that flaxseed may inhibit development of cardiovascular diseases, diabetes mellitus, inflammatory bowel disease, and polycystic ovary syndrome. These targets have an association with various stages of the diseases. The ingestion of 15 g of raw ground golden flaxseed containing complex carbohydrates before breakfast decreases the 2-h postprandial glycemic response in men with T2DM (Moreira et al., 2022). Flaxseeds have antihyperglycemic properties without severe reported side effects. Many health benefits of flaxseed protein hydrolysates, such as anti-hypertension ability, antibacterial activity, antioxidant capacity, anti-diabetic ability, and the inhibition ability of calmodulin-dependent neuronal nitric oxide synthase, have been reported (Franck et al., 2019; Logarušić et al., 2020; Nwachukwu and Aluko, 2018a; Nwachukwu and Aluko, 2018b; Perreault et al., 2017; Wu et al., 2019) previously. Flaxseed yogurt is useful not only in women with PCOS but also for women during menopause (Tang, 2019a; Tang, 2019b).

## Conclusion

As flaxseed is widely used as a medicinal herb in many countries and is an affordable dietary supplement which is used by individuals with T2DM to improve glycemic control, we studied the physicochemical parameters and toxicity measures of flaxseed (α-linolenic acid) by computational methods and bioinformatics tools. The results predicted that flaxseed is a biologically active medicinal plant. Flaxseed is safe to use in mutagenicity, tumorigenicity, irritation, and reproductive effect, while it is unsafe for use in hERG toxicity and cardiac failure. The use of flaxseed is not recommended for pregnant women. The docking results showed good binding affinities for the three potential (CCDC28b–flaxseed), (PDCD6IP–flaxseed), and (USP34–flaxseed) interactions. The GO analysis for the three target–flaxseed complexes shows heart looping, heart morphogenesis and heart development, vascular endothelial growth factor receptor-2 signaling pathway, negative regulation of biological process, negative regulation of phospholipase A2 activity, oxygen-containing compound, and regulation of fat cell differentiation, which is related to cardiovascular, cancer, diabetes mellitus, and other related diseases. The PPI analysis shows that flaxseed can be used for anti-cholesterol, antioxidant, anti-tumor, and antihyperglycemic activities. Flaxseed is also useful to serve the basic pathophysiological theory of IBD, which involves immune dysregulation, barrier defects, and microbial dysregulation. The results indicate that flaxseed can be used as an option to treat PCOS, which may be influenced by environmental factors, prenatal hormone imbalance, lifestyle, and genetic abnormalities. Therefore, more preclinical trials and clinical trials of a longer duration are needed to identify extensive bioactive components from flaxseed.

## Data Availability

The original contributions presented in the study are included in the article/Supplementary Material; further inquiries can be directed to the corresponding author.
